# Supplementary material for: Dysferlin-mediated phosphatidylserine sorting engages macrophages in sarcolemma repair
Source: Nat Commun. 2016 Sep 19;7:12875. doi: 10.1038/ncomms12875 (PMC5031802; doi:10.1038/ncomms12875)
Supplement: Supplementary Information — Supplementary Figures 1-7, Supplementary Tables 1-2, Supplementary Note 1, Supplementary Methods and Supplementary References. [file ncomms12875-s1.pdf]

Supplementary Figures

Supplementary Figure 1

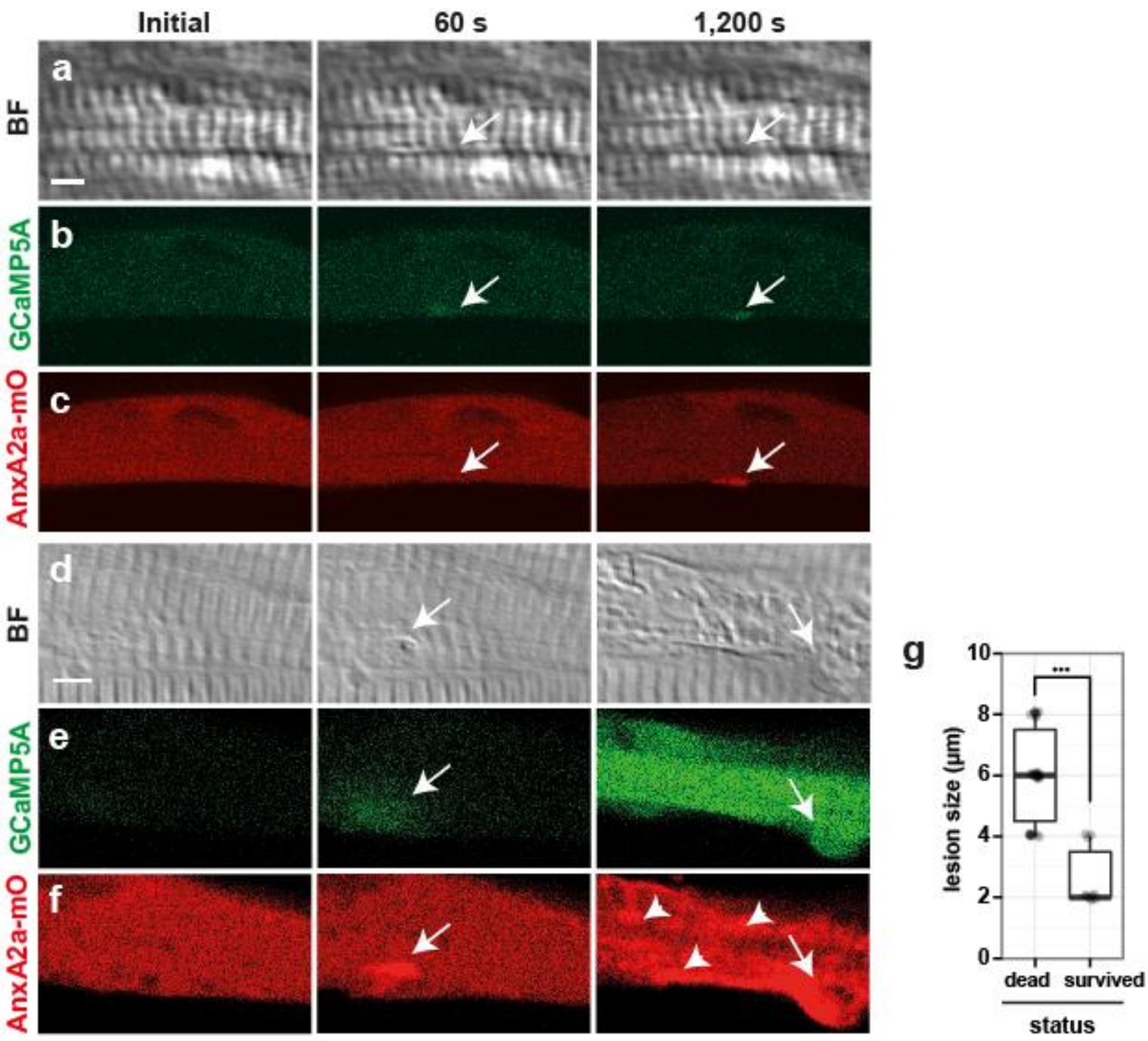

Supplementary Figure 1 | Fate of myofibers depends on size of membrane wound.

a-f, Myofibers with a lesion (arrows) of either ≤4 µm (a-c) or ≥4 µm (d-f) were analyzed for repair patch formation (AnxA2a-mO, c, f), local Ca^{2+} changes (GCaMP5A, b, e) and corresponding bright field views (BF, a, d), prior to (initial), 60 s and 1,200 s after wounding. Immediately after wounding, GCaMP5A was locally activated regardless of the lesion size (b, e). Ca^{2+} levels at lesions of ≤4 µm size resolved back to baseline after 1,200 s with the exception of Ca^{2+} in the repair patch

(b), however, lesions ≥4 μm led to propagation of a Ca^{2+} wave across the entire cell (e), frequently followed by sliding of the repair patch along the myofiber (arrow, d-f), and precipitation of AnxA2a-mO to internal membranes of myofibers (f, arrowheads), a hallmark of cell death. **g,** Survival rate of myofibers was analyzed as a function of initial lesion size (Y-axis in μm). Myofibers with lesions ≥4 μm underwent cell death (left column; n=30), while cells with lesions ≤4 μm survived (right column: n=10; Student's t-test, p<0.001). Scale bar: 4 μm.

Supplementary Figure 2

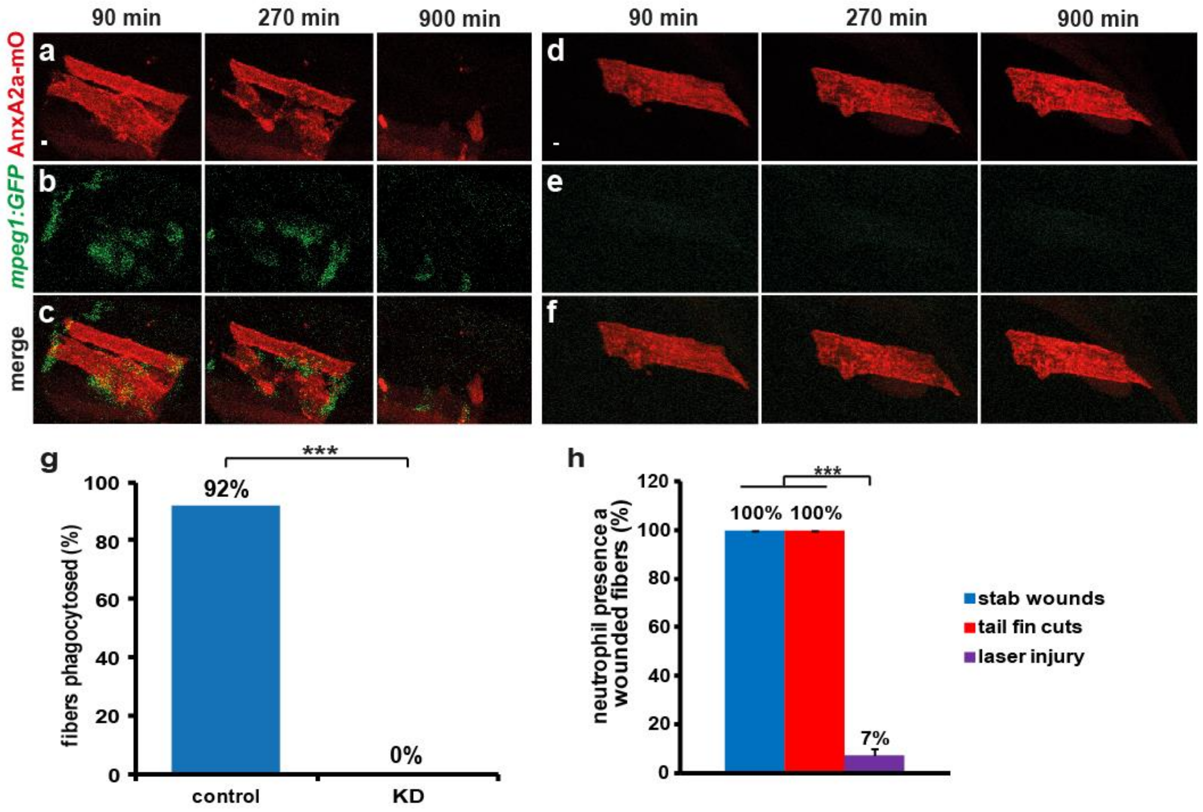

Supplementary Figure 2 | Macrophages remove severely damaged myofibers. a-f, Dead myofibers show precipitation of AnxA2a-mO (red) to internal membranes (a and d). In controls dead myofibers were removed by attracted macrophages (b, green) within 900 min after wounding (c). In morphants dead myofibers persisted beyond imaging time (d and f), due to the absence of macrophages (e). Panels a-b and d-e were merged in c and f, respectively. **g**, Macrophages remove dead myofibers. In controls (left, n=12), 92% of dead myofibers were removed by macrophages within 20 h of observation. In contrast, removal of dead cells (right, n=20) was totally abolished (0%) in animals lacking macrophages. **h**, Neutrophils were barely recruited to wounds inflicted with a laser (purple, n=126) while stab wounds caused by insertion of a glass needle into the somitic musculature (blue, n=23) and cutting of the tail fin (red, n=12) attracted neutrophils in all cases. Thus infection and concomitant differences in inflammatory status may have a bearing on

which immune cells become engaged. Data was analyzed as maximum projections. p-values were calculated according to Fisher’s exact test, $p<0.001$. Scale bars: 4 µm.

Supplementary Figure 3

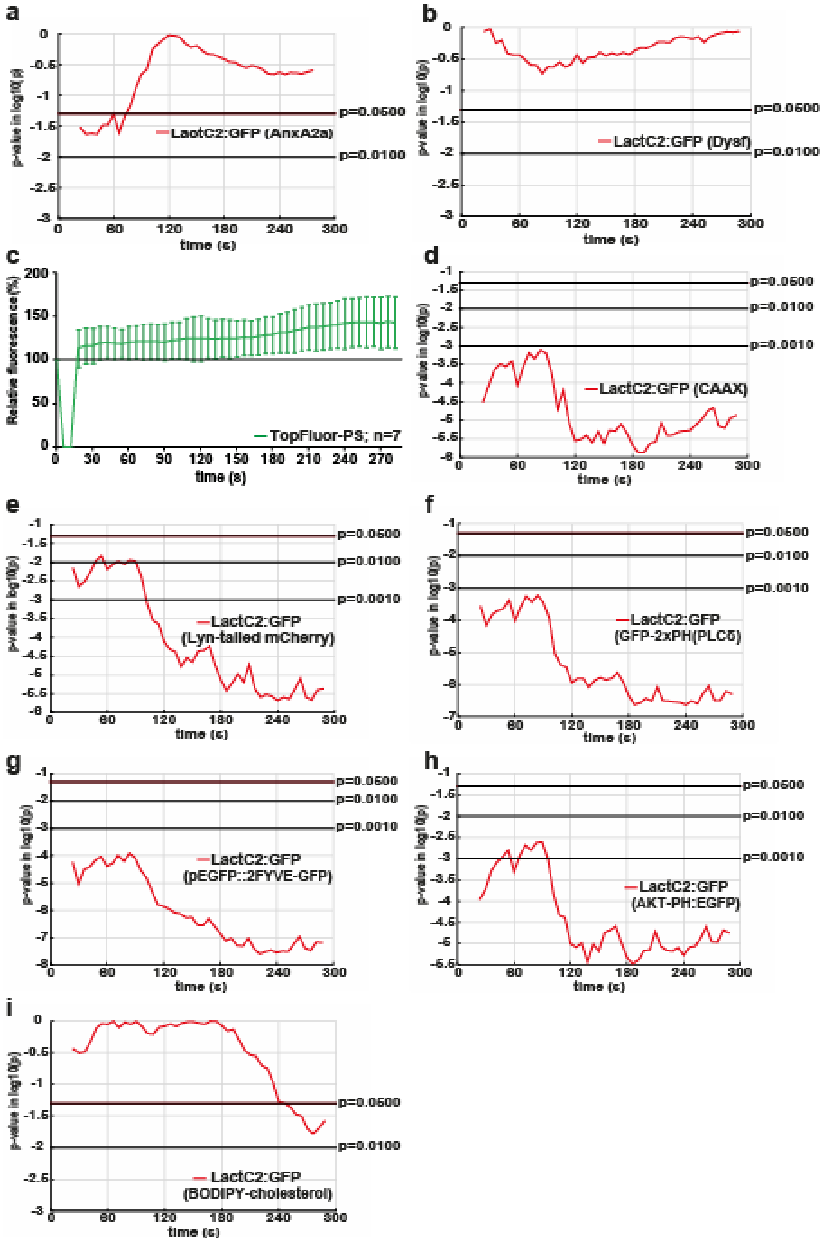

Supplementary Figure 3 | TopFluor-PS accumulation and significance blots.

a, LactC2:GFP accumulated significantly faster during the first 60 s after lesioning compared to AnxA2a-mO. **b**, There is no significant difference between LactC2:GFP and Dysf-mO enrichment at the wounded site. **c**, Rapid accumulation of TopFluor-PS

(green) after inflicting membrane wounds. **d-i**, The PS-sensor LactC2:GFP piled up significantly more compared to the common membrane markers CAAX-mCherry (d) and Lyn-tailed mCherry (e) and lipid markers such as GFP-2xPH(PLC δ) (f), pEGFP::2FYVE-GFP (g) and AKT-PH:EGFP (h). No significant difference in the enrichment of LactC2:GFP and BODIPY-cholesterol at the lesion site throughout the first 240 s were noted while significant differences in accumulation intensity were observed for the last 60 s (i). Significance was tested using MATLAB software. In charts (b-i) the two sided Welch's t-Test with Bonferroni correction was performed. Note: In charts (e-i) the same data set for LactC2:GFP accumulation (Fig. 2c, green) was used for statistical comparison.

Supplementary Figure 4

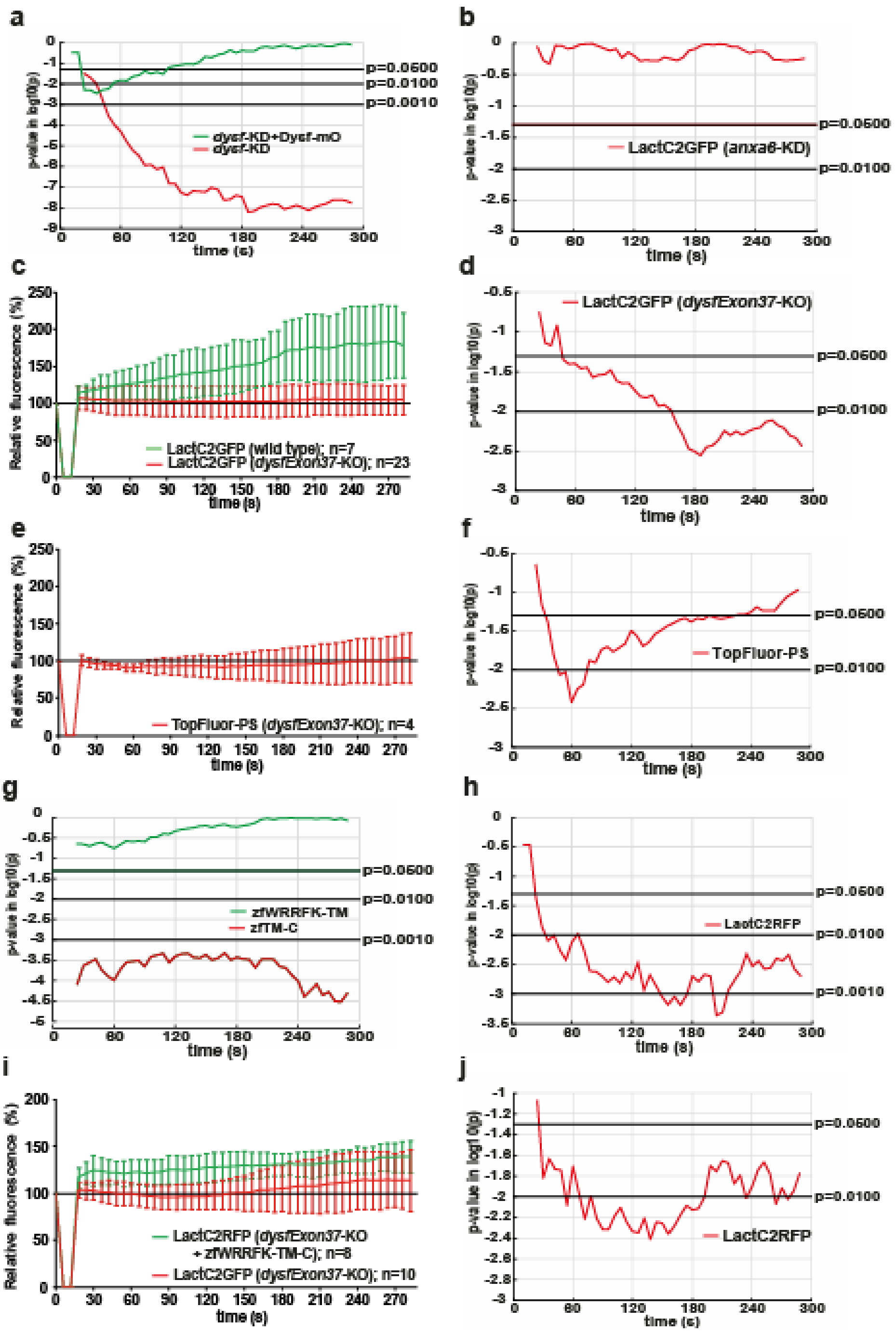

Supplementary Figure 4 | Dysf is crucial for PS accumulation, while the motif WRRFK is necessary for Dysf accumulation.

a, Significant differences in accumulation of PS at the site of membrane damage in *dysf*-KD injected fish compared to controls (red), while in rescues (*dysf*-KD+Dysf-mO) PS enrichment is significantly increased during the first 100 s and then converges to the control (green). **b**, No significant disparity in PS concentration at membrane wounds in *anxa6* morphants compared to controls (red). **c-d**, *dysfExon37*-KO embryos were analyzed for LactC2:GFP accumulation (c, red) and significance was tested (d) against wild type siblings (c, green). **e-f**, *dysfExon37*-KO embryos were injected with TopFluor-PS and accumulation was quantified (e) and tested for significance (f) against wild type siblings shown in Figure S2C. **g**, While the C-terminal mutant construct (zfWRRFK-TM) shows no significant difference to the wild type control construct (zfWRRFK-TM-C, green), the mutant construct lacking the WRRFK motif (zfTM-C) exhibits significant loss of PS replenishment within the repair patch (red). **h**, PS accumulation (LactC2:RFP) was significantly reduced (red) in *dysf*-KD fish co-injected with zfTM-C compared to co-injection with zfWRRFK-TM-C. **i-j**, For rescue experiments *dysfExon37*-KO embryos were co-injected with LactC2:RFP and zfWRRFK-TM-C and PS enrichment was quantified. In charts (c, e, i), the change of fluorescence at the site of lesion is indicated as percentage relative to the undamaged state (black) ± standard deviation, n≥4. Significance was tested using MATLAB software. In charts (a-b, d, f-h and j) the two sided Welch's t-Test with Bonferroni correction was performed.

Supplementary Figure 5

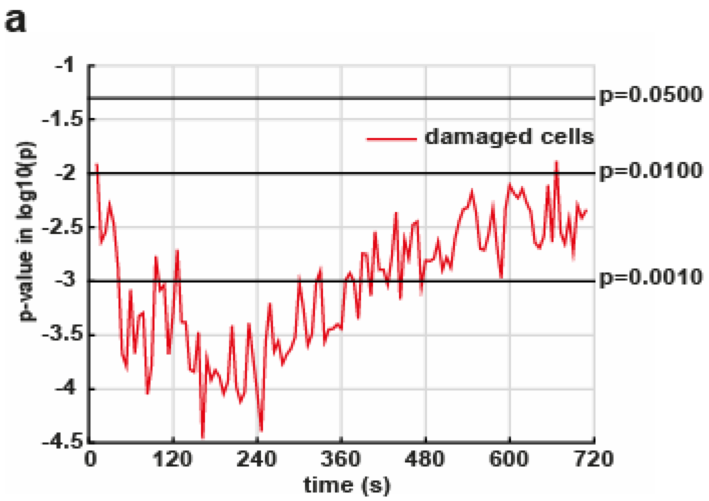

Supplementary Figure 5 | Dysf is recruited to membrane lesions from the adjacent sarcolemma.

a, Significance blot of FLIP experiments. (n=8). Significance was tested using MATLAB software and performing the two sided Welch's t-Test with Bonferroni correction.

Supplementary Figure 6

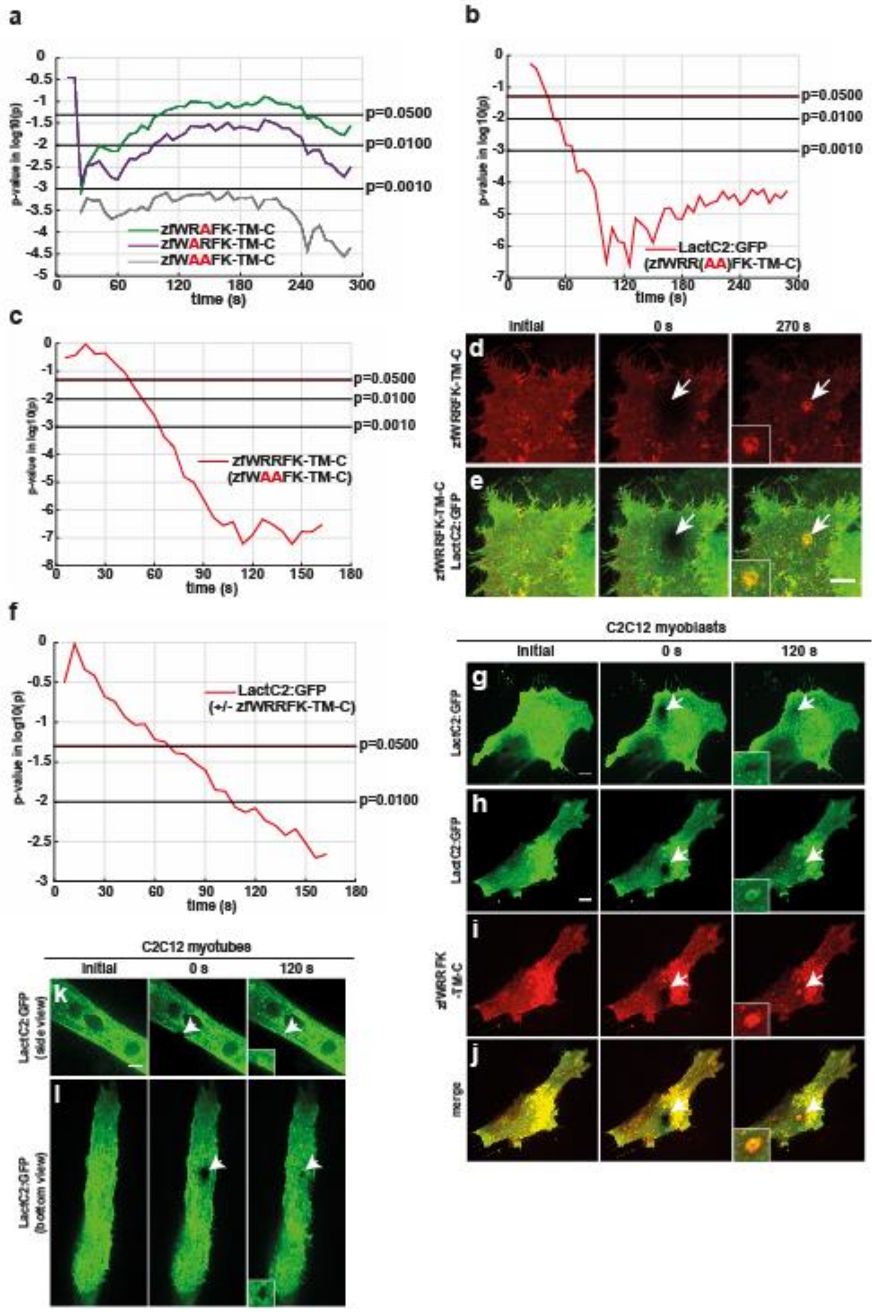

Supplementary Figure 6 | Two arginines within the zfWRRFK motif of Dysf are essential for PS and Dysf accumulation.

a, Significance was tested comparing wild type constructs (zfWRRFK-TM-C) with single arginine to alanin substituted mutant constructs zfWRAFK-TM-C (green) and

zfWARFK-TM-C (purple). The latter shows significant differences to the control throughout the whole imaging time, whereas zfWRAFK-TM-C showed significant differences in the first 100 s and turned again significant after 240 s. A double alanin mutant construct (zfWAAFK-TM-C, grey) showed significant disparity to the control throughout the whole imaging time. **b**, Significance was tested for LactC2:GFP enrichment at the lesion between co-injections of LactC2:GFP and zfWRRFK-TM-C or LactC2:GFP and zfWAAFK-TM-C. Significant disparity can be seen from 40 s until the end of imaging (red). **c**, Significance blot of HeLa cells either transfected with zfWAAFK-TM-C or zfWRRFK-TM-C. The latter accumulates significantly more from 45 s onwards (red). **d-e**, HeLa cells were co-transfected with LactC2:GFP (see Fig. 5g) and zfWRRFK-TM-C-mCherry. The damaged site is indicated by white arrows. Cells were imaged initially , directly after damage (0 s) and after 270 s. Accumulation of zfWRRFK-TM-C-mCherry was observed (d, inlay) and co-localization with LactC2:GFP is indicated by the yellow repair patch (e, inlay). Panels in e are merged images of d and Fig. 5g. **f**, After 70 s enrichment of PS at the membrane wound in co-transfected (LactC2:GFP and zfWRRFK-TM-C) cells differs significantly from single transfected (LactC2:GFP) cells (red). **g-j**, undifferentiated C2C12 myoblasts transfected with LactC2:GFP alone and imaged before 0 s (arrow) and 120 s after lesioning (arrow) showed no PS accumulation but only baseline recovery (i, arrow, inset). In the presence of zfWRRFK-TM-C, LactC2:GFP accumulated in the repair patch (j-l, arrows, insets). **k-l**, differentiated C2C12 myotubes (k, side view; l, bottom view) expressing LactC2:GFP only and imaged before 0 s (arrow) and 120 s after lesioning (arrow) showed PS enrichment at the lesion site (i, arrow, inset). Note: Experiments used to test significance (zfWRRFK-TM-C) in chart a are the same as used in Supplementary Fig. 3g. Significance was tested using MATLAB software. In

charts (a-c and f) the two sided Welch's t-Test with Bonferroni correction was performed. Scale bars: 10 µm.

Supplementary Figure 7

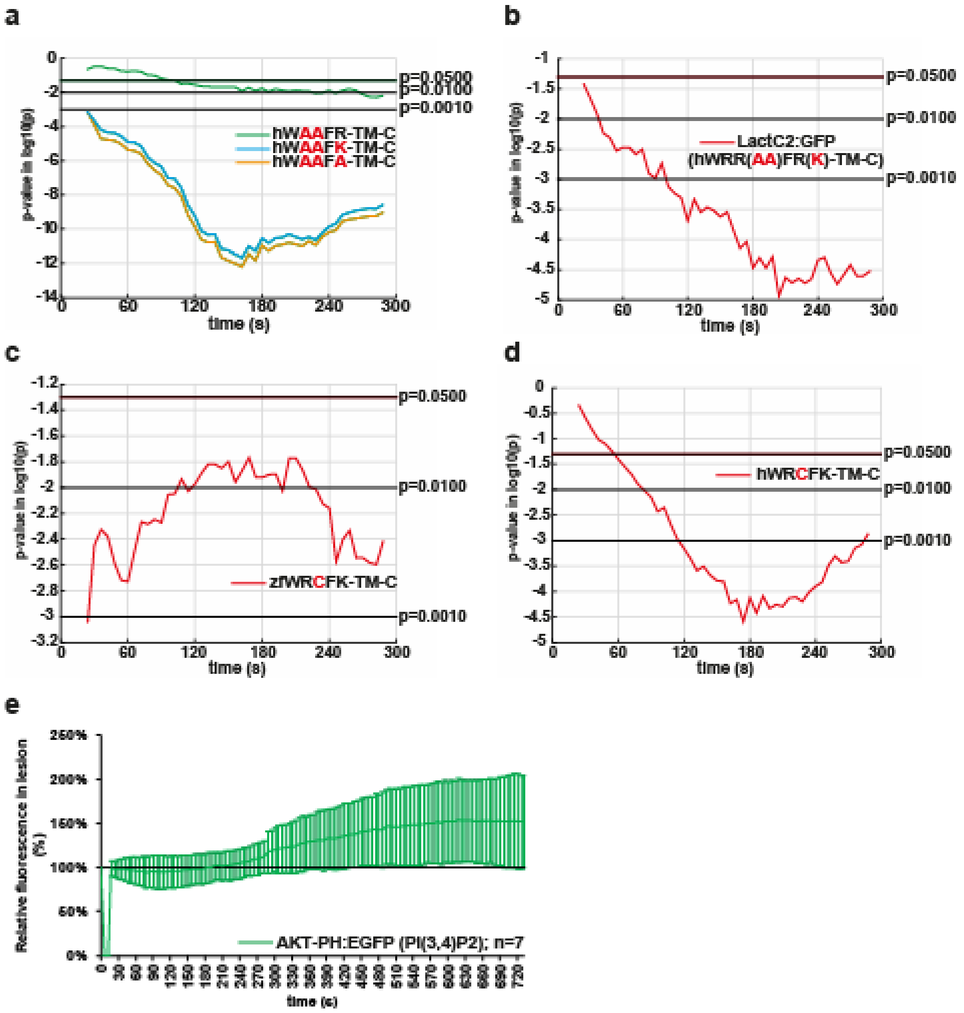

Supplementary Figure 7 | Human dysferlin harbors a conserved arginine-rich motif that mediates accumulation of PS in the lesion patch.

a, hWRRFR-TM-C shows significant differences in accumulation from 18 s compared to hWAAFK-TM-C (blue) and hWAAFA-TM-C (yellow) and from 90 s to hWAAFR-TM-C (green). **b**, Significant disparity between PS accumulation in morphants co-injected with hWRRFK-TM-C compared to morphants co-injected with hWAAFK-TM-C (red). **c**, Significant difference in Dysf accumulation comparing wild type zfWRRFK-TM-C and the pathogenic R2042C mutation zfWRCFK-TM-C (red). **d**, Significant

difference in Dysf accumulation comparing wild type hWRRFR-TM-C and the pathogenic R2042C mutation hWRCFR-TM-C (red). **e**, AKT-PH:EGFP marking PI(3,4)P2 accumulates slowly in 720 s post wounding. In e, the change of fluorescence at the lesion is indicated as percentage relative to the undamaged state ± standard deviation. Note: Experiments for fWRRFR-TM-C used to test for significance are the same in a and d. Similar, experiments used to check significance in chart c are the same as in Supplementary Fig. 4g, 6a. Significance was tested using MATLAB software. In charts a-d the two sided Welch's t-Test with Bonferroni correction was performed.

Supplementary Table 1

Primer	Sequence (5' to 3' direction)	Comments
**Fusion vectors**		
**F.LinkNxhoI**	GCGCTCGAGGCCGGTGGCGGAGGGTCTGGAG	used to clone the Linker for N-terminal fusion into the pME backbone
**R.LinkNclaI**	TTATCGATGGCTGATCCACCCCCGCCG	
**F.CherryN.XhoI**	CGACTCGAGGGCGCCACCATGGTGAGCAAGGGCGAGGAG	used to make N-terminal fusion constructs for mCherry in the pME backbone
**R.CherryN.Link.ClaI**	GCGATCGATGCCTGATCCACCCCCGCCGGAACCGCCCCCTCCAGACCCTCCGCCACCC TTGTACAGCTCGTCCATG	
**F.CloC.Link.SpeI**	CGAACTAGTGGCGGTGGCGGAGGGTCTGGAGGGGGCGGT TCCGGCGGGGGTGGATCAGTGAGCAAGGGCGAGGAGCTGTTCAC	used to make C-terminal fusion constructs
**R.CloC.SacI**	GCGGAGCTCGCCCTAGCATTTAGGTGACACTATAGAATAG	

		for Clover in the pME backbone
**F.EosFPNkpnI**	GCCAGGTACCCCGCCACCATGAGTGCGATTAAGCCAGACATG	used to make N-terminal fusion constructs for mEosFP in the pME backbone
**R.EosFPNxhoI**	GCCACTCGAGGGCCCGTCTGGCATTGTCAGG	
**cloning of cDNAs**		
**F.LactC2GFP.ClaI**	GCATCGATGCGGCCACCATGGTGAGCAAGGGCGAG	used to subclone LactC2:GFP into the pME backbone
**R.LactC2GFP.XhoI**	CGACTCGAGGGCCTAACAGCCCAGCAGCTCCAC	
**F.LactC2N.ClaI**	GCATCGATGCCTGCACTGAACCCCTAGGCCTG	used to subclone LactC2 into N-terminal fusion vectors into the pME backbone
**R.LactC2N.SpeI**	GCACTAGTGGCCTAACAGCCCAGCAGCTCCAC	
**F.shTM-DysfC.ClaI**	GCGATCGATGCCACCATGTGGCTTATTCTGGGCCTCTTTATAC	used to clone zfTM-C into the pME backbone
**R.shTM-DysfC.SpeI**	GCCACTAGTGGCCTGTGTTCCCTTTCCTAG	
**F.shTM-DysfN.ClaI**	GCGATCGATGCCTGGCTTATTCTGGGCCTCTTTATAC	used to clone zfTM-N into the pME backbone
**R.shTM-DysfN.SpeI**	GCCACTAGTGGCTCACTGTGTTCCCTTTCCTAG	
**F.h.shTM-**	CGGGTACCGCCACCATGTGGGCCATCATCCTCTTC	used to

**Do.KpnI**		clone hTM-C into the pME backbone
**R.h.shTM-Do.SpeI**	GCACTAGTGCCGCTGAAGGGCTTCACCAGC	
**F.unc45b195BamHI**	CAGAGGATCCGCGCTTAATGGTTTCTTACAGTA	used to subclone unc45b.195 promoter into the p5E backbone
**R.unc45b195SacII**	GCTGCCGCGGGCGATAGGGTCTATTTATGGAG	
**F.CAAXEosFPNClaI**	GCCAATCGATGCCAAGCTGAACCCTCCTGATGA	used to fuse CAAX to mEosFP N-terminally into the pME backbone
**R.CAAXEosFPNSpeI**	GCGACTAGTGGCTCAGGAGAGCACACACTTGC	
**F.secA5.ClaI**	GCATCGATGGCGCCACCATGCATAAGGTTTTGCTGGCAC	used to subclone sek.AnxaV-YFP into the pME backbone
**R.secA5.XhoI**	GCCTCGAGGGCTTACTTGTACAGCTCGTCCATGC	
**F.AKT-PH.ClaI**	GCATCGATGGCAACGACGTAGCCATTGTGAAG	used to subclone AKT-PH:EGFP into the pCS2+ backbone
**R.AKT-PH.SpeI**	GCACTAGTGGCTTATCTAGATGAATTCCATG	
**F.FYVE.GFP.BamHI**	GCCGGATCCCGGCCACCATGGTGAGCAAGGGCGAGG	used to subclone 2FYVE-GFP into the pME backbone
**R.FYVE.GFP.XhoI**	GGCCTCGAGGCTTATCCTTGCAAGTCATTG	
**F.PCLd2xPH.BamHI**	GCCGGATCCCGGCCACCATGGACTCGGGCCGGGACTTC	used to subclone

**R.PCLd2xPH.Xhol**	GGCCTCGAGGCTTACTTGTACAGCTCGTCCATG	GFP-2×PH(PLCδ) into the pME backbone
**F.Lyn-Tailed-mCh-flu.KpnI**	ATGGTACCGCCGCCACCATGGGATGTATTAAATCAAAAAG	used to subclone Lyn-Tailed-mCherry-luorin into the pME backbone
**R.Lyn-Tailed-mCh-flu.ClaI**	GCATCGATGGCTTATTTGTATAGTTCATCCATG	
**F.Ca-Report.HindIII**	GGCCAAGCTTGCCACCATGGGTTCTCATCATCATC	used to subclone GCaMP5A: GFP into the pME backbone
**R.Ca-Report.BamHI**	GCGGGATCCGCCTTACTTCGCTGTCATCATTTG	
**mutagenesis**		
**F.Clo.KpnI-mut**	AGGACGACGGTACGTACAAGACCCGCG	was used to mutate a KpnI site within Clover (silent mutation)
**R.Clo.KpnI-mut**	CGCGGGTCTTGTACGTACCGTCGTCCT	
**F.unc195.del**	CCTCTCCATAAATAGACCCTATCACAAGTTTGTACAAAAAAGTTGAAC	used to delete a short sequence 3' of the unc45b.195 within the p5E
**R.unc195.del**	GTTCAACTTTTTTGTACAAACTTGTGATAGGGTCTATTTATGGAGAGG	
**zebrafish *dysferlin* mutants**		
**F.shTM-Do-**	TATCGATGCCACCATGTGGAGGAGGTTTAAGTGGCTTATTCTGGGCC	used to

**Dysf_RR_inser**		clone
**R.shTM-Do-Dysf_RR_inser**	GGCCCAGAATAAGCCACTTAAACCTCCTCCACATGGTGGCATCGATA	zfWRRFK-TM-C
**F.shTM-CloC+AA**	TATCGATGCCACCATGTGGGCGGCGTTTAAGTGGCTTATTCTGGGCC	used to clone
**R.shTM-CloC+AA**	GGCCCAGAATAAGCCACTTAAACGCCGCCCACATGGTGGCATCGATA	zfWAAFK-TM-C
**F.shTM-CloC+RA**	TATCGATGCCACCATGTGGAGGGCGTTTAAGTGGCTTATTCTGGGCC	used to clone
**R.shTM-CloC+RA**	GGCCCAGAATAAGCCACTTAAACGCCCTCCACATGGTGGCATCGATA	zfWRAFK-TM-C
**F.shTM.RRtoAR**	CGTTTAGGTTCATTGTGTGGGCGAGGTTTAAGTGGCTTATTC	used to clone
**R.shTM.RRtoAR**	GCAAATCCAAGTAACACACCCGCTCCAAATTCACCGAATAAG	zfWARFK-TM-C
**F.zfshTM-R2043C**	TATCGATGCCACCATGTGGAGGTGTTTTAAGTGGCTTATTCTGGGCC	used to clone
**R.zfshTM-R2043C**	GGCCCAGAATAAGCCACTTAAAACACCTCCACATGGTGGCATCGATA	zfWRCFK-TM-C
**F.shTM-CloDelC-term**	TAACTATGCTGCTATGGCCACTAGTGGCGGTG	used to clone
**R.shTM-CloDelC-term**	CACCGCCACTAGTGGCCATAGCAGCATAGTTA	zfWRRFK-TM
**human dysferlin mutants**		
**F.h.shTM-Do+RR**	GGTACCGCCACCATGTGGCGGCGTTTCCGGTGGGCCATCATCCTC	used to clone
**R.h.shTM-Do+RR**	GAGGATGATGGCCCACCGGAAACGCCGCCACATGGTGGCGGTACC	hWRRFR-TM-C
**F.hshTM-R2042C**	GAGGATGATGGCCCACCGGAAACACCGCCACATGGTGGCGGTACC	used to clone
**R.hshTM-R2042C**	GGTACCGCCACCATGTGGCGGTGTTTCCGGTGGGCCATCATCCTC	hWRCFR-TM-C
**F.h.shTM-Do+AA**	GGTACCGCCACCATGTGGGCGGCTTTCCGGTGGGCCATCATCCTC	used to clone
**R.h.shTM-Do+AA**	GAGGATGATGGCCCACCGGAAAGCCGCCCACATGGTGGCGGTACC	hWAAFR-TM-C

**F.h.shTM-Do+MAAFK**	GGTACCGCCACCATGTGGGCGGCGTTCAAGTGGGCCATCATCCTC	used to clone hWAAFK-TM-C
**R.h.shTM-Do+MAAFK**	GAGGATGATGGCCCACTTGAACGCCGCCCACATGGTGGCGGTACC	
**F.h.shTM-Do+MAAFA**	GGTACCGCCACCATGTGGGCGGCTTTCGCGTGGGCCATCATCCTC	used to clone hWAAFA-TM-C
**R.h.shTM-Do+MAAFA**	GAGGATGATGGCCCACGCGAAAGCCGCCCACATGGTGGCGGTACC	
**Morpholinos**		
i36/e37	GGCAATCGCTGAAGAGAGTGCAGAA	splice MO to knockdown *dysferlin* (final 0.8 µM)
***i36/e37* contr.**	GGCAAACCCTGAACAGACTCCAGAA	5 bp mismatch control MO (final 0.8 µM)
**mo_*anxa6***	TCCTGCCTCATACAGCTCCTATAAT	splice MO to knockdown *annexin6* (final 0.3 µM)
**mo_*anxa6* contr.**	TCGTCCCTGATACACCTCGTATAAT	5 bp mismatch control MO (final 0.3 µM)
pu.1	GATATACTGATACTCCATTGGTGGT	used to knockdown *pu.1* (final 0.5 µM)
gcsfr	GAAGCACAAGCGAGACGGATGCCAT	used to knockdown *gcsfr* (final

		0.5 µM)
irf8	AATGTTTCGCTTACTTTGAAAATGG	used to knockdown *irf8* (final 0.6 µM)
**contr. MO for *pu.1, gcsfr, irf8***	GTTAAGCTATTCTTATTCACGCATT	control MO (concentration depending on how many MOs were co-injected)
**Crispr/Cas9**		
**guideRNA (*DysfExon37*)**	GGTGGAATGTGGCGGATCTG	sequence of the targeting gRNA without PAM
**T7guideRNA-oligo (*DysfExon37*)**	TAATACGACTCACTATA-GGTGGAATGTGGCGGATCTG-GTTTTAGAGCTAGAAATAGCAAG	T7-sequence of the targeting gRNA without PAM-overlapping sequence to constant oligo
**STOP-oligo (*DysfExon37*)**	TCAGGTGGAATGTGGCGGAT-GTCATGGCGTTTAAACCTT AATTAAGCTGTTGTAG-CTGTGGTTCAGTCGTGCGTC	injected together with Cas9-protein (Life technologies) and gRNA

**Constant oligo**	AAAAGCACCGACTCGGTGCCACTTTTTCAAGTTGATAACG GACTAGCCTTATTTTAACTTGCTATTTCTAGCTCTAAAAC	annealed with T7guideRNA-oligo to make cloneless gRNA
**Left-primer (*DysfExon37*)**	GCCTCATGTGACCTTTTAACCT	Primer used for genotyping
**Right-primer (*DysfExon37*)**	GGCATGCTGTAACAACACATTT	
**WT-primer (*DysfExon37*)**	GGAATGTGGCGGATCTGTGG	
**guideRNA (*irf8*)**	GCGGTCGCAGACTGAAACAG	sequence of the targeting gRNA without PAM
**T7guideRNA-oligo (*irf8*)**	TAATACGACTCACTATA-GCGGTCGCAGACTGAAACAG-GTTTTAGAGCTAGAAATAGCAAG	T7-sequence of the targeting gRNA without PAM-overlapping sequence to constant oligo
**STOP-oligo (*irf8*)**	CGGGCGGTCGCAGACTGAAAGTCATGGCGTTTAAACCTTAATTAAGCTGTTGTAGCAGTGGCTTATAGAACAGAT	injected together with Cas9-protein (Life technologies) and gRNA
**Left-primer**	GGCAACATAAGGCGTAGAGAT	Primer used

(*irf8*)		for genotyping
**Right-primer (*irf8*)**	TTTCGCTTACTTTGAAAATGGAC	
**For squencing (*irf8*)**	TTTCGCTTACTTTGAAAATGGA	Primer used to amplify the mutant region to send it for sequencing

Supplementary Table 2

Sensor	Description	Structure/molecule detected	References
AnxA2a-mO	AnnexinA2a tagged with mOrange	Repair patch, internal membranes of dying cells	This study
LactC2:GFP	Genetically encoded fusion of C2-domain of human LactAdherin with GFP	PS	20
LactC2:RFP	Genetically encoded fusion of C2-domain of human LactAdherin with RFP	PS	20
LactC2:mEosFP	Genetically encoded fuson of C2-domain of human LactAdherin with photoswitchable mEosFP	PS	This study
Dysf-mO	Fragment of Dysferlin tagged with mOrange	Dysferlin, repair patch	This study
CAAX-mCherry	Genetically encoded prenylation motif fused to mCherry	Cell membranes	22
CAAX-mEosFP	Genetically encoded prenylation motif fused to photoswitchable mEosFP	Cell membranes	This study
Lyn-tailed mCherry	Genetically encoded fusion of Lyn-tailed membrane targeting domain of the Src-family kinase Lyn with mCherry	Cell membrane	23
GFP-2×PH(PLCδ)	Genetically encoded lipid sensor	PI(4,5)P2)	24
pEGFP::2FYVE–GFP	Genetically encoded lipid sensor	PIP3	25
AKT-PH:EGFP	Genetically encoded lipid sensor	PI(3,4)P2)	26
secA5-YFP	A secreted form of human AnnexinA5 fused to YFP	Extracellular PS	28
zfWXXFK-TM-C	Fragment of zebrafish Dysferlin fused to Clover, mCherry or photoswitchable mEosFP	Dysferlin, repair patch	This study

hWXXFX-TM-C	Fragment of human Dysferlin fused to Clover or mCherry	Dysferlin, repair patch	This study
GCaMP5A	Genetically encoded calcium sensor	Ca^{2+} ions	13
BODIPY-cholesterol	23-(dipyrromethene boron difluoride)-24-norcholesterol, synthetic fluorescent cholesterol	Distribution of cholesterol	Avanti lipids
TopFluor PS	1-palmitoyl-2-(dipyrromethene boron difluoride)undecanoyl-sn-glycero-3-phospho-L-serine, synthetic fluorescent PS	Distribution of PS	Avanti lipids
mpeg1:GFP	Transgene driving GFP	macrophages	14
LysC:dsRED	Transgene driving RFP	neutrophils	15

Supplementary notes

US and GUN were funded by HGF, DFG (STR 439/8-1, Ni 291/12-1), EC IP ZF-HEALTH and BMBF-MIE. In addition, this research work is part of the project „Molecular Interaction Engineering: From Nature's Toolbox to Hybrid Technical Systems“, which is funded by the German Federal Ministry of Education and Research (BMBF), funding code 031A095 C.

Supplementary methods

Cloning. All cloning was carried out following standard procedures[1] and the instructions of the suppliers of the used enzymes and kits as indicated below. Primers for cloning (Supplementary Table 1) were purchased from Metabion (Germany, Planegg). PCR to amplify small inserts (<700 bp) was performed using GoTaq or GoTaq2 polymerase (Promega, Germany, Mannheim or Fermentas, Germany, Darmstadt), whereas longer PCR products were amplified with Pfu polymerase (Promega, Germany, Mannheim) using a gradient-PCR protocol (50°C - 70°C annealing temperature). The elongation time was chosen according to the insert length (GoTaq: 1000 bp/min; Pfu: 500 bp/min). PCR products were separated on an ethidium bromide/agarose gel, excised and purified with a gel purification kit

(Peqlab, Germany, Erlangen) followed by restriction enzyme digestion of inserts and backbone vectors (NEB Germany, Frankfurt am Main and Fermentas, Germany, Darmstadt) at 37°C for 1 h. After digestion, vectors and inserts were again gel purified as described above. The vectors were incubated with thermosensitive alkaline phosphatase (Fermentas, Germany, Darmstadt) for 1 h at 37°C and again gel purified. Ligation was set at a ratio of 3:1 insert to vector and incubated at 16°C over night with T4 DNA Ligase (Promega, Germany, Mannheim) followed by transformation into *E.coli Xl1blue* or *Top10*.

Expression vectors for fusion proteins were cloned with the Gateway system and LR clonase II kit (Invitrogen, Germany, Darmstadt). The *unc45b* promoter (from -505 to -310 relative to the ATG of *unc45b9*) was subcloned into the 5' entry vector (*p5E*) between BamHI and SacII sites[2] for muscle specific expression. For HeLa cell experiments the CMV promoter of the p5E vector was used (Tol2kit #382)[3]. All cDNAs fused either N- or C-terminally, spaced by a linker sequence, to fluorescent proteins were subcloned into the middle entry vector (*pME*). For C-terminal fusion constructs, *mEosFP*[4, 5], *Clover*[6] or *mCherry*[3] were PCR amplified and inserted within the MCS of the *pME* vector between SpeI and SacI sites. For N-terminal fusion, *mEosFP* was inserted within KpnI and XhoI sites, whereas for *mCherry,* XhoI and ClaI sites were used. *Clover* was inserted between the XhoI site and checked for correct orientation after successful integration. A linker sequence (3xGly-Gly-Gly-Gly-Ser) was used to space cDNAs coding for the desired proteins and the fluorescent protein. For C-terminal fusion, the linker sequence was integrated into the primer, whereas for N-terminal fusion, the linker was separately inserted into the *pME* backbone using XhoI and ClaI. cDNAs were cloned to fluorescent proteins N- or C-terminally according to published data[7, 8]. All truncated versions of zebrafish (ClaI/SpeI) and human (KpnI/SpeI) dysferlin were tagged C-terminally except for

mEosFP fusions, which were tagged N-terminally between ClaI/SpeI sites. *LactAdherinC2* was either amplified via PCR including *EGFP* and inserted into the *pME* backbone between ClaI and XhoI sites, or only *LactAdherinC2* was amplified and fused N-terminally to *mEosFP* and *mCherry* using ClaI and SpeI sites. *CAAX* was amplified and fused N-terminally to *mEosFP* between ClaI/SpeI sites. Human *secreted AnxA5-YFP* was inserted into the *pME* backbone using ClaI/XhoI sites[9]. *GFP-2×PH(PLCδ)* and *pEGFP::2FYVE–GFP* were inserted into the *pME* vector by using BamHI and XhoI sites, whereas *AKT-PH:EGFP* was cloned into the *unc45b-pCS2+* backbone between ClaI and SpeI sites[7, 10, 11, 12, 13]. *GCaMP5A:GFP* was fully cloned into the *pME* vector using HindIII and BamHI sites[14]. All plasmids were sequenced. *p5E* and *pME* vectors were transformed into *Xl1blue E.coli* and plated on LB + kanamycin plates. The desired promoter within the *p5E*, the fusion protein within the *pME*, the *p3E-polyA* (Tol2kit #302) and the *pDEST* (Tol2kit #394) were recombined according to the manual of the LR clonase II kit (Invitrogen, Germany, Darmstadt). The reaction was incubated over night at room temperature. *Top10 E.coli* were transformed and plated on LB + ampicillin plates. Plasmid DNA was prepared following the instructions of the supplier of the reagents (Qiagen, Germany, Hilden). For primer sequences, see Supplementary Table 1.

Site directed mutagenesis. Expression constructs (zfTM-C:CloFP, zfTM-C:mCherry, mEosFP:zfTM-C, hTM-C:CloFP, hTM-C:mCherry) were modified by site-directed mutagenesis using mutagenesis-PCR. 1 µl template DNA (100 ng/µl), 1 µl Pfu-Polymerase, 1 µl dNTP-Mix (10 mM), 1 µl Primer-Mix (forward + reverse, each 1:20 diluted from stock) and 5 µl 10x Pfu-Buffer; added up to 50 µl with H_2O. Primers were designed using an online tool, QuikChange Primer Design:

http://www.genomics.agilent.com/primerDesignProgram.jsp.

The following program was used for PCR : 5 min 95°C, 1 min 95°C, 1 min 54°C, 16-25 min 72°C (12 cycles), 1 min 40°C, 30 s 72°C, hold at 4°C. Next, the template DNA was fragmented by DpnI digestion for 3 h at 37°C. After confirming successful PCR on an agarose gel, 5 µl of the PCR reaction was used for bacterial transformation (Top10 *E. coli*). Two to three colonies were picked, DNA was prepared and sent for sequencing. For primer sequences, see Supplementary Table 1.

Microinjections. The collected eggs were cleaned and approximately 100 were used for each injection. The eggs were transferred onto a 10 cm petridish lid and water was removed. Embryos were injected (Eppendorf FemtoJet) through the chorion directly into the yolk during the 1-2 cell stage. Injections were carried out with purified plasmid DNA (20-80 ng/µl; midiprep, Qiagen) diluted in dH_2O supplemented with 0.01% phenol red. One drop (~1/10 of the yolk size) was injected per embryo. Injection needles were pulled from borosilicate glass capillary tubes with filament (0.58 mm diameter, Warner Instruments) using a micropipette puller (Sutter Instruments). Injected embryos were grown to 3-5 dpf at 28°C.

Morpholinos. Morpholinos (GeneTools, LLC) against *dysferlin* mRNA (0.8 mM), *anxa6* mRNA (0.3 mM) and 5 bp mismatch control morpholinos (GeneTools, LLC) at the same concentration as targeting morpholinos were used as described previously[7]. Morpholinos (GeneTools, LLC) for knocking down macrophage and neutrophil populations were used as described[15, 16, 17]. Briefly, mRNA coding for *pu1*, *gcsfr* and *irf8* were targeted with morpholinos as a triple injection at final concentrations of 0.5 mM, 0.5 mM and 0.6 mM, respectively, supplemented with 0.01% phenol red. For each injection a drop the size of 1/10 of the yolk volume was injected. The control morpholino was used at the same concentration (max. 1.7 mM) as the targeting morpholinos. For sequences of the morpholinos and control morpholinos, see Supplementary Table 1.

***Dysferlin* and *irf8* knock-out using Crispr/Cas9.** *Dysferlin Exon37* and *irf8* knock-out mutants were generated by the CRISPR/Cas9 technique as previously published[18]. Briefly, *dysferlin Exon37* and *irf8 Exon 1* was targeted by a CHOPCHOP (https://chopchop.rc.fas.harvard.edu/) designed guideRNA (Supplementary Table 1), which was injected at a concentration of 200 ng/µl together with a STOP oligo (final concentration 3 µM; Supplementary Table 1) for insertion into the cutting site and Cas9-mRNA nanos (200 ng/µl)[19] into AB_2O_2 wild type fish. After 24 h, 10 random embryos were sacrificed and genomic DNA was extracted using the HotShot method (remove water, add 100 µl 50 mM NaOH per embryo, 20 min 95°C, hold at 4°C, add 10 µl 1 M Tris HCl pH 7.5). PCR with a gene specific (left primer; Supplementary Table 1) and a stop cassette specific primer (right primer; Supplementary Table 1) was performed to identify positive embryos. If positive embryos were identified, the rest of the batch was raised as F0 generation. F0 offspring (24 hpf, 8 embryos per couple, 4 embryos pooled) was screened by PCR for germ line transmission with the same primers as described above (Supplementary Table 1). Identified batches were raised and the F1 generation identified by tail fin cuts. Genomic DNA was extracted (HotShot) and PCR was performed with a left and right primer giving a ~400 bp fragment. PCR products were gel-purified, sub-cloned into the pGEM-T Easy backbone (Promega) and sent for sequencing. Mutant F1 fish were incrossed to obtain homozygous mutant embryos that were injected with, e.g., LactC2:GFP in the one cell stage. 3-5 dpf embryos were embedded into 0.5% low melting point (LMP) agarose for damaging experiments. Subsequently, embryos were cut free from LMP agarose and genomic DNA was extracted (HotShot) for each embryo. Embryos were identified by genotyping PCR. All primers used are listed in Supplementary Table 1.

Lipid detection. Genetically encoded sensors were injected as plasmids into the zygote[8, 12, 20, 21]. BODIPY-cholesterol (Avanti Polar Lipids, Inc.) was injected as

previously published[22]. TopFluor-PS (Avanti Polar Lipids, Inc.) was dissolved in DMSO (1-10 mM) and injected into the yolk of 1-2 cell stage embryos (20–40 pmol per embryo). BODIPY-cholesterol injected embryos were analyzed at 5 dpf; TopFluor-PS injected embryos were imaged at 3-5 dpf.

Imaging. 3- to 5-day-old larvae were prepared as previously published [7]. By using a hair-forceps (a hair is taped to a 100 µl pipette tip), the embryos were embedded in 0.5% low melting point agarose supplemented with 0.02% MESAB and positioned onto a microscopy glass slide. By using a confocal microscope (TCS SP2, Leica Microsystems, Mannheim, Germany), the sarcolemma of single cells was damaged at 32x zoom with a Ti:Sa laser (MaiTai, Spectra Physics, Mountain View, CA) set to 860 nm (gain 65%, offset 26%) using the region-of-interest (ROI) set to 0.25-2 μm^2. Two scans (4x line alignment) were performed. For *in vivo* time lapse imaging, time intervals were set to 6 s for a total of 45 or 120 frames using 8x zoom. For larger muscle damage (7.5 μm^2), full frame scanning (32x zoom; 2x line alignment) was performed twice.

For long term time lapse imaging (16-22 h) *in vivo* under a Leica TCS SP5, embryos were embedded in LMP agarose (0.5%) in a 5 cm petridish and covered with 10 ml E3 medium (60x stock: 5 mM NaCl, 0.17 mM KCl, 10 mM HEPES, 0.33 mM $MgSO_4 \cdot 7H_2O$, 0.33 mM $CaCl_2 \cdot 6H_2O$) containing 0.02% MESAB and 0.003% phenylthiourea (PTU). Fibers were damaged on the Leica TCS SP2 as described above. Multiple positions of the damaged cells (max. 2 damaged cells/embryo) were imaged under a Leica TCS SP5 upright microscope (HCX PL APO 20x/0.70 lambda blue IMM CORR or HCX APO L 40x/0.80 W U-V-I objectives) using the bright field and two fluorescence channels (488 nm/561 nm) in parallel scanning mode. Z-stacks covering 40-90 µm were acquired every 2 µm at each position (10-30 positions in total). Each position was imaged every 5 min for 16-22 h in 512x512 pixel format with

a depth of 8-bit by bidirectional resonant scanning at 8 kHz. Images with an extended depth of field were generated in ImageJ[23] through maximum projections for the temporal analysis of the fluorescence from the tagged molecules of interest.

Quantification of macrophages and repair patch removal. To assess knockdown efficiency, leukocytes recruited to muscle injury were counted manually in controls or morphants.

To quantify the removal of the repair patch by macrophages, Tg(*mpeg1:GFP*) fish were co-injected with AnxA2a-mO together with a control morpholino or a cocktail of three published morpholinos to reduce leukocyte populations [15, 16, 17]. Then single cells (small damage) were damaged as described above. Imaging times at the Leica SP2 were set at least to 1 h, whereas, at the SP5, imaging times were set to a maximum of 22 h. The movies were analyzed as maximum projections.

To investigate whether macrophages are necessary to remove dead muscle cells, larger laser lesions (7.5 µm²) were introduced into myofibers of morphants and control fish as described above. The wounded area was imaged for max. 20 h. Macrophage recruitment toward damaged cells was counted manually.

Quantification of the lesion size. Small lesions were induced as described above. The lesion was followed for at least 60 min using the 561 nm channel (RFP) and the bright field channel. The size of the lesion was measured and estimated for the first 7 min by using the scale bar function within ImageJ. As soon as AnxA2a-mO precipitated toward internal membranes, the cell was considered as dead and the measurement was stopped.

Detection and quenching of phosphatidylserine by externally supplied secA5-YFP. For detection and quenching of phosphatidylserine by externally supplied secA5-YFP, a plasmid encoding human secA5-YFP was injected as described above. The sarcolemma of non-expressing cells were damaged and imaged (every 6

s for 45 frames) using the bright field and YFP (514 nm) channels. For saturation experiments, Tg(*mpeg1:GFP*) fish were injected with a plasmid encoding secA5-YFP. The "scanning-time" of recruited macrophages described by Rosenberg and colleagues[24] was quantified by measuring the time between the macrophage arrival and the first observed phagocytosis.

Tailfin cuts and stabwounding. 3-5 days old embryos were embedded in 1.5 % low melting point agarose in a 5 cm petridish. The tailfin was cut with a scalpel or embryos were stabbed through the agarose into the somitic musculature with a glass needle (injection needle). Embryos were covered with water supplemented with 0.02% MESAB immediately and neutrophils were counted after 2h.

Software, statistics and image manipulation. For each protein or lipid response, at least 5 independently damaged cells were examined. ImageJ was used for quantification of the fluorescence at the wounded area. Images were stacked to movies. For each experiment, a circular area was drawn on top of the damaged area adjusted to the size of the lesion. The mean intensity was calculated as a function of time (total 48 frames) and values copied to Microsoft Excel 2007. The first frame corresponds to the undamaged cell, the second and third frames were damaging scans with the pulsed infrared laser (MaiTai), while the other 45 frames are time lapse images (6 s interval) of the wounded cell. The mean intensity of the encircled area of the first frame for each experiment was set to 100%. The other 47 frames were normalized to the first frame by dividing the encircled mean intensity with the mean intensity of the first frame. Means and standard deviations were calculated by Excel 2007 for each experimental data set. ImageJ was used for manual quantification of all experiments and Excel 2007 for statistics. Long-term imaging movies were analyzed with Extended Depth of Field (EDF). Depending on the data sets p-values were calculated using Fisher's exact test or for kinetic analysis, with

Welch's Test followed by Bonferroni correction using MATLAB (The Mathworks, Natick, MA). Experiments that showed drastic focal drift in z-axis as well as myofibers that showed signs of cell death, e.g. movement of the patch out of frame, secondary contraction or AnxA2a-mO precipitation to internal membranes, were excluded from quantifications. Basic image processing such as cropping, brightness, contrast and color merge was performed using Adobe Illustrator or ImageJ.

Super-resolution localization microscopy. Super-resolved images of zebrafish were acquired with a modified inverted microscope (Axiovert 200, Zeiss) equipped with a high N.A. water immersion objective (C-Apochromat, 63x, N.A. 1.2, Zeiss). Three solid-state lasers were employed, with wavelengths 561 nm (Cobolt Jive, Cobolt, Solna, Sweden), 473 nm (LSR473-200-T00, Laserlight, Berlin, Germany) and 405 nm (Stradus 405-100, Vortran, Sacramento, CA) for fluorescence excitation and cell membrane damaging. The laser beams were combined via dichroic mirrors (AHF, Tübingen, Germany) and guided through an acousto-optic tunable filter (AOTFnC-400.650, A-A, Opto-Electronic, Orsay Cedex, France) to control the laser intensities. The photoactivatable protein mEosFP*thermo* was converted from its green to its red emitting form by using low intensity (0 – 50 W/cm²) 405 nm light and excited by simultaneous 561 nm illumination (200 – 400 W/cm²). After passing through the excitation dichroic filter (z 405/473/561/635, AHF, Tübingen, Germany), the fluorescence emission was filtered by a 607/50 band-pass filter (AHF, Tübingen, Germany) and recorded with a back-illuminated EMCCD camera (Ixon Ultra 897, Andor, Belfast, Northern Ireland) at 30 ms time resolution. After 405 nm laser damage, strong 561 nm laser irradiation (800 – 1,200 W/cm²) was used to bleach the activated mEosFP*thermo* fluorophores in the red channel for about 1 min. This time is also needed so that the damaged fiber stabilizes again for the following imaging process. 1,500 – 2,000 camera frames were acquired for further analysis, starting 1 –

2 min after 405 nm laser-induced membrane damage. Fibers which displayed severe membrane shrinkage or other morphological changes were excluded from imaging.

Data analysis for super-resolution imaging. Data of localization microscopy were analyzed using custom written analysis software, a-livePALM[25] running under the MATLAB R2014a (The Mathworks, Natick, MA) environment. The molecule identification thresholding parameter (*P*-value) was adjusted to 0.04 – 0.1 for different image conditions to identify molecules for further fitting. The software was run on a personal computer using an Intel Core i7-4930K processor clocked at 3.40 GHz with 32.0 GB memory and a NVIDIA GeForce GTX 760 graphics card.

Trajectories of single molecules were acquired using MATLAB code made available by John C. Crocker and colleagues (http://www.physics.emory.edu/faculty/weeks//idl/). To link molecules from adjacent frames, their maximum displacement between successive camera frames was set to 400 nm; the minimal trajectory length was set to 4 frames. By limiting the photon threshold value, 130 ± 5 of typically 500 trajectories were selected for each measurement and further analyzed within the measured region 2 – 15 µm away from the damage site. For each individual trajectory, its projection onto a line connecting the midpoint of the trajectory with the lesion site was calculated (Fig. 4d). The resulting displacement was divided by the number of steps in the trajectory, yielding positive (toward the lesion) or negative speeds (away from the lesion). A parameter, R_{proj}, was defined as the ratio of the summed magnitudes of all positive and negative speeds within one measurement; it is greater (smaller) than one for net movement toward (away from) the lesion, and equal to one for random motion. For each measurement, the R_{proj} parameter was computed based on 130 ± 5 local trajectories and the user-specified damage site, using custom-written MATLAB programs.

Supplementary references

1. Sambrook J, Russell DW. *The condensed protocols from Molecular cloning : a laboratory manual*. Cold Spring Harbor Laboratory Press (2006).

2. Etard C, Armant O, Roostalu U, Gourain V, Ferg M, Strahle U. Loss of function of myosin chaperones triggers Hsf1-mediated transcriptional response in skeletal muscle cells. *Genome Biol* **16**, 267 (2015).

3. Kwan KM*, et al.* The Tol2kit: a multisite gateway-based construction kit for Tol2 transposon transgenesis constructs. *Dev Dyn* **236**, 3088-3099 (2007).

4. Wiedenmann J*, et al.* EosFP, a fluorescent marker protein with UV-inducible green-to-red fluorescence conversion. *Proc Natl Acad Sci U S A* **101**, 15905-15910 (2004).

5. Nienhaus K, Nienhaus GU, Wiedenmann J, Nar H. Structural basis for photo-induced protein cleavage and green-to-red conversion of fluorescent protein EosFP. *Proc Natl Acad Sci U S A* **102**, 9156-9159 (2005).

6. Lam AJ*, et al.* Improving FRET dynamic range with bright green and red fluorescent proteins. *Nat Methods* **9**, 1005-1012 (2012).

7. Roostalu U, Strahle U. In vivo imaging of molecular interactions at damaged sarcolemma. *Dev Cell* **22**, 515-529 (2012).

8. Yeung T, Gilbert GE, Shi J, Silvius J, Kapus A, Grinstein S. Membrane phosphatidylserine regulates surface charge and protein localization. *Science* **319**, 210-213 (2008).

9. van Ham TJ, Mapes J, Kokel D, Peterson RT. Live imaging of apoptotic cells in zebrafish. *Faseb J* **24**, 4336-4342 (2010).

10. Stauffer TP, Ahn S, Meyer T. Receptor-induced transient reduction in plasma membrane PtdIns(4,5)P2 concentration monitored in living cells. *Curr Biol* **8**, 343-346 (1998).

11. Marshall JG*, et al.* Restricted accumulation of phosphatidylinositol 3-kinase products in a plasmalemmal subdomain during Fc gamma receptor-mediated phagocytosis. *J Cell Biol* **153**, 1369-1380 (2001).

12. Vieira OV*, et al.* Distinct roles of class I and class III phosphatidylinositol 3-kinases in phagosome formation and maturation. *J Cell Biol* **155**, 19-25 (2001).

13. Mason D*, et al.* Alteration of epithelial structure and function associated with PtdIns(4,5)P2 degradation by a bacterial phosphatase. *J Gen Physiol* **129**, 267-283 (2007).

14. Akerboom J*, et al.* Optimization of a GCaMP calcium indicator for neural activity imaging. *J Neurosci* **32**, 13819-13840 (2012).

15. Rhodes J, *et al.* Interplay of pu.1 and gata1 determines myelo-erythroid progenitor cell fate in zebrafish. *Dev Cell* **8**, 97-108 (2005).

16. Li L, Jin H, Xu J, Shi Y, Wen Z. Irf8 regulates macrophage versus neutrophil fate during zebrafish primitive myelopoiesis. *Blood* **117**, 1359-1369 (2011).

17. Liongue C, Hall CJ, O'Connell BA, Crosier P, Ward AC. Zebrafish granulocyte colony-stimulating factor receptor signaling promotes myelopoiesis and myeloid cell migration. *Blood* **113**, 2535-2546 (2009).

18. Gagnon JA, *et al.* Efficient mutagenesis by Cas9 protein-mediated oligonucleotide insertion and large-scale assessment of single-guide RNAs. *PLoS One* **9**, e98186 (2014).

19. Moreno-Mateos MA, *et al.* CRISPRscan: designing highly efficient sgRNAs for CRISPR-Cas9 targeting in vivo. *Nat Methods* **12**, 982-988 (2015).

20. Fairn GD, Hermansson M, Somerharju P, Grinstein S. Phosphatidylserine is polarized and required for proper Cdc42 localization and for development of cell polarity. *Nat Cell Biol* **13**, 1424-1430 (2011).

21. Kwon Y, Hofmann T, Montell C. Integration of phosphoinositide- and calmodulin-mediated regulation of TRPC6. *Mol Cell* **25**, 491-503 (2007).

22. Holtta-Vuori M, *et al.* BODIPY-cholesterol: a new tool to visualize sterol trafficking in living cells and organisms. *Traffic* **9**, 1839-1849 (2008).

23. Schindelin J, *et al.* Fiji: an open-source platform for biological-image analysis. *Nat Methods* **9**, 676-682 (2012).

24. Rosenberg AF, Wolman MA, Franzini-Armstrong C, Granato M. In vivo nerve-macrophage interactions following peripheral nerve injury. *J Neurosci* **32**, 3898-3909 (2012).

25. Li Y, Ishitsuka Y, Hedde PN, Nienhaus GU. Fast and efficient molecule detection in localization-based super-resolution microscopy by parallel adaptive histogram equalization. *ACS Nano* **7**, 5207-5214 (2013).
